# Deposition of Copper on Poly(Lactide) Non-Woven Fabrics by Magnetron Sputtering—Fabrication of New Multi-Functional, Antimicrobial Composite Materials

**DOI:** 10.3390/ma13183971

**Published:** 2020-09-08

**Authors:** Marcin H. Kudzin, Zdzisława Mrozińska, Anna Kaczmarek, Agnieszka Lisiak-Kucińska

**Affiliations:** Lukasiewicz Research Network—Textile Research Institute, Brzezinska 5/15, 92-103 Lodz, Poland; zmrozinska@iw.lodz.pl (Z.M.); akaczmarek@iw.lodz.pl (A.K.); alisiak@iw.lodz.pl (A.L.-K.)

**Keywords:** poly(lactide), nonwovens, melt-blown, antimicrobial material, polyamides, copper, magnetron sputtering, antibacterial activity, composite

## Abstract

The paper presents the method of synthesis; physico-technical and biological characterization of a new composite material (PLA–Cu^0^) obtained by sputter deposition of copper on melt-blown poly(lactide) (PLA) non-woven fabrics. The analysis of these biofunctionalized non-woven fabrics included: ultraviolet/visible (UV/VIS) transmittance; scanning electron microscopy/energy-dispersive spectroscopy (SEM/EDS); attenuated total reflection Fourier transform infrared (ATR-FTIR) spectroscopy; ability to block UV radiation; filtration parameters (air permeability); and tensile testing. The functionalized non-woven composite materials were subjected to antimicrobial tests against colonies of Gram-negative (*Escherichia coli*), Gram-positive (*Staphylococcus aureus*) bacteria and antifungal tests against the *Chaetomium globosum* fungal mould species. The antibacterial and antifungal activity of PLA–Cu^0^ suggests potential applications as an antimicrobial material.

## 1. Introduction

Poly(lactide) (PLA) is a multifunctional application polymer widely engaged in various biomedical applications, including controlled drug delivery, wound healing, tissue engineering and regenerative medicine [1,2,3,4,5,6,7,8].

Substantial importance in this field is played by PLA composites equipped with antibacterial additivities, in the majority organic bactericides (e.g., ampicillin [9] chlorhexidine [10,11], ciprofloxacin [12,13], doxycyclin [14], gentamycin [15], oflaxin and vancomycin [16], triclosan [17,18,19], and/or curcumin [20], etc.). Due to an established role of metal nanoparticles in medicine [21,22,23,24,25,26,27,28,29,30,31,32,33], the group of increasing importance is PLA-inorganic hybrids [34], due to their high antibacterial efficiency, simplicity and also low cost of technological preparation.

Some of the representative applications of antibacterial PLA composites are listed in Table 1.

Among various inorganic bactericides of medicinal interest, considerable attention has been focused on copper and its salts reflected by over 5000 documents on antibacterial copper and nearly 300 documents on antibacterial metallic copper abstracted by Scopus [34,35], respectively.

In the light of continuous expansion of antibiotic resistance to bacteria, copper nanoparticles (CuNPs) with such attributes of copper as chemical stability (E_1_ = 0.52 V) [36], developed surface and antibacterial prolonged antibacterial activity [37] (a very slow dissolution of metallic copper in water with subsequent formation of very poor soluble copper oxides Cu_2_O/CuO [37]), as well as a fair abrasion resistance of copper layer’s (hardness 230 kgf mm^−2^) [38], contrasted with their negligible contact toxicity toward animals [39,40,41,42], became a valuable alternative to traditional antibiotics [25,27,43,44]. The antibacterial activity of copper’s metallic surface is regarded as resulting from two supplemental mechanisms—surface–surface interaction of copper and bacteria (contact killing) and/or surface oxidation of copper with subsequent release of antibacterial cupric ions [25,27,43,44,45,46,47,48].

Polymer–Cu–nanoparticles, being the convenient platform for metallic, antibacterial copper have been formed by a wide array of methods, including chemical, biological synthesis methods, and physical methods [49,50,51,52,53], including the magnetic sputtering method [54,55]. This method exhibits an especially convenient character—it is simple and ecofriendly, allowing deposition of the required amount of deposited metal in function of the time applied.

As a part of our experimentation program focused on phosphonic acids [56,57,58,59] and textile chemistry of their hybrids with a polymer matrix [60,61], we present the preparation, and characterization of a new multifunctional, biodegradable composite material, PLA–Cu, composite. This composite was obtained by a surface modification of melt-blown poly(lactide) non-wovens with copper, using the direct current (DC) magnetron sputtering method.

## 2. Materials and Methods

### 2.1. Materials

**Polymers**

- Poly(lactic) acid, type Ingeo™ Biopolymer 3251D was purchased from NatureWorks LLC (Minnetonka, MN, USA).

**Bacteria subtype**

- *Staphylococcus aureus* (ATCC 6538);
- *Escherchia coli* (ATCC 25922).

**Fungal subtype**

- *Chaetomium globosum* (ATCC 6205), bacterial strains and fungal strains from Microbiologics (St. Cloud, MN, USA).

### 2.2. Methods

#### 2.2.1. Melt-Blown Technique, Fabrication of Poly(lactide) (PLA) Non-Wovens

Nonwovens samples were manufactured by the melt-blown technique using a laboratory extruder (Axon, Limmared, Sweden) [60]. Samples of nonwoven fabrics were manufactured in the form of a sheet. Processing parameters for fabrication of Poly(lactic) samples are listed in Table 2.

#### 2.2.2. Magnetron Sputtering Modification of PLA Non-Wovens

The obtained poly(lactide) non-wovens were modified using a DC magnetron sputtering system produced by P.P.H. Jolex s. c. (Czestochowa, Poland). The copper target (Testbourne Ltd., Basingstoke, UK) with 99.99% purity was used. The distance between the target and the substrate was equal to 15 cm. The deposition of coatings was carried out in the atmosphere of argon. In order to optimize the process and avoid the destruction of the PLA substrates, different powers were applied to the target from 350 up to 1000 W. Finally, the following parameters were applied for modification: the power discharge of 700 W, with the resulting power density equal to 0.72 W/cm^2^ and the working pressure of 2.0 × 10^−3^ mbar. In order to vary the copper content, two different deposition times were applied, i.e., 10 min (sample name/assignment: PLA–Cu^0^(10)) and 30 min (sample name: PLA–Cu^0^(30)). Sputtered sample size was: 60 cm × 20 cm.

#### 2.2.3. Scanning Electron Microscopy/Energy-Dispersive Spectroscopy (SEM/EDS)

The microscopic analysis of fibers was performed on a Tescan Vega 3 scanning electron microscope (Brno, Czech Republic) [60]. Magnification was from 500× to 5000×.

#### 2.2.4. Attenuated Total Reflection Fourier Transform Infrared Spectroscopy (ATR-FTIR)

The chemical surface structure of PLA samples was assessed using ATR-FTIR Jasco’s 4200 (Tokyo, Japan) spectrometer, with Pike Gladi ATR attachment (Cottonwood, AZ, USA), in the range: 400–4000 cm^−1^.

#### 2.2.5. Ultraviolet-Visible (UV-VIS) Spectroscopy and Determination of the Protective Properties against UV Radiation

Physical properties as transmittance [%T] of modified PLA samples were assessed using a Jasco V-550 spectrophotometer (Tokyo, Japan), in the range: 200–800 nm. The same apparatus was used to determine the ultraviolet protection factor (UPF) of samples, according to the standard EN 13758-1:2002 *Textiles. Solar UV protective properties. Method of test for apparel* fabrics [62], on the basis of Equation (1):

(1) $$UPF=\frac{\int_{290}^{400}E\left(\lambda \right)\varepsilon \left(\lambda \right)d\left(\lambda \right)}{\int_{290}^{400}E\left(\lambda \right)\varepsilon \left(\lambda \right)T\left(\lambda \right)d\left(\lambda \right)}$$

where:

*Δλ*—the wavelength interval of the measurements;
*ε(λ)*—the erythema action spectrum, measure of the harmfulness of UV radiation for human skin;
*E(λ)*—the solar irradiance;
*T(λ)*—the spectral transmittance at wavelength *λ*.

The UPF value of the samples was determined as the arithmetic mean of the UPF values for each of the samples, reduced by the statistical value depending on the number of measurements performed, at a confidence interval of 95%.

#### 2.2.6. Filtration Properties

Air permeability of PLA samples was tested according to EN ISO 9237:1998 [63], analogously as samples of PP [59] and/or PLA [60] nonwovens. An FX 3300 Textest AG (Klimatest, Wroclaw, Poland) permeability tester was used.

#### 2.2.7. Tensile Properties

Tensile testing of poly(lactic acid) samples was performed according with the EN ISO 10319:2015 standard [64]. Stretching speed was 20 mm/min. An Tinius Olsen H50KS (Horsham, PA, USA) tester was used.

#### 2.2.8. Atomic Absorption Spectrometry with Flame Excitation (FAAS)

Determination of copper content in poly(lactic acid) non-woven fabrics samples was assessed using a single-module Magnum II microwave mineralizer from Ertec (Wroclaw, Poland) and Thermo Scientific Thermo Solar M6 (LabWrench, Midland, ON, Canada) atomic absorption spectrometer equipped with a 100 mm titanium burner, coded lamps with a single-element hollow cathode, background correction: D2 deuterium lamp.

The total copper content of the sample M [mg/kg; ppm] was calculated according to Equation (2) [65]:

(2) $$M=\frac{Ci\;x\;V\;}{mi}\;\left[\frac{\mathrm{mg}\;}{kg}\right]$$

where:

*C*_i_—metal concentration in the tested solution [mg/L];
*m_i_*—mass of the mineralized sample [g];
*V*—volume of the sample solution [mL].

#### 2.2.9. Antibacterial Tests

The PLA–Cu^0^ fabrics anti-bacterial activity was tested according to standard: PN-EN ISO 20645:2006 [66], against a colony of Gram-positive bacteria: *Staphylococcus aureus* (ATCC 6538) and Gram-negative bacteria: *Escherchia coli* (ATCC 25922) analogously as in PP non-wovens [60]. Antibacterial activity of samples was tested by agar diffusion method using Muller Hinton medium agar [67,68].

#### 2.2.10. Antifungal Activity

The antifungal activity of resulting samples was tested according to PN–EN 14119:2005 [69]. The standard indicate tests of antifungal activity on a *Chaetomium globosum* (ATCC 6205), analogously as in PP nonwovens [60].

## 3. Results and Discussion

### 3.1. SEM/EDS

**Figure 1:**
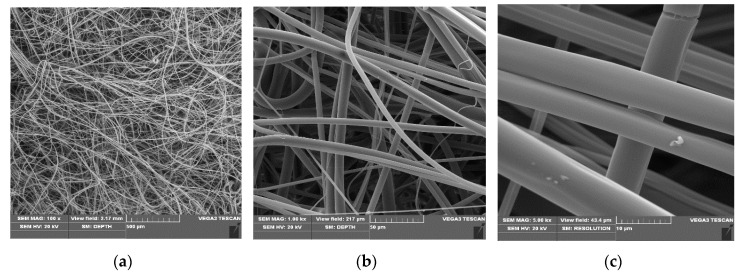
Scanning electron microscopy (SEM) images of poly(lactide) (PLA) non-woven, magnification: (**a**) 100×, (**b**) 1000×, (**c**) 5000×.

**Figure 2:**
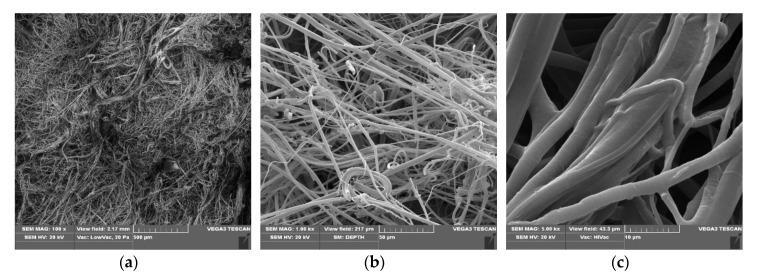
SEM images of PLA–Cu^0^(10), magnification: (**a**) 100×, (**b**) 1000×, (**c**) 5000×.

**Figure 3:**
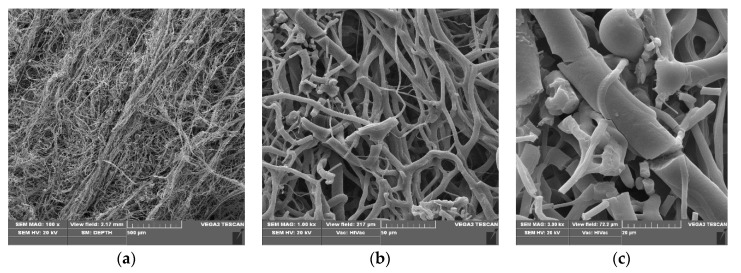
SEM images of PLA–Cu^0^(30), magnification: (**a**) 100×, (**b**) 1000×, (**c**) 3000×.

SEM micrographs of poly(lactide) samples and modified poly(lactide) samples coated with copper (PLA–Cu^0^(t)) are presented in Figure 1, Figure 2 and Figure 3, respectively.

The SEM images of the starting PLA non-woven and PLA–Cu^0^(t) composites reflect the changes in their morphology occurring during sputtering process (PLA→PLA–Cu^0^(10)→PLA–Cu^0^(30)). Thus, PLA fibres images present uniform randomly oriented fibers, with interconnected pores and space with relatively smooth surface in PLA fibre images (Figure 1). The diameters of poly(lactide) fibers applied ranged: 0.65–5.0 µm (Figure 1c).

PLA–Cu^0^(10) composites images (Figure 2) show uniform randomly oriented fibers (Figure 2b,c) and visible layer of copper on the surface fibers (Figure 2c). On the other hand, PLA–Cu^0^(30) composite images also present uniform randomly oriented fibers (Figure 3a), but with high fibers crack content (Figure 3b,c). the PLA–Cu0(30) sample shows a substantial contribution (20–30%) of shorter fragments (length: 10 to 30 µm; diameter: 2 to 13 µm), without sharp edges, Cu covered. Fiber fracture and damage of the PLA–Cu^0^(30) composite during copper sputtering of PLA in 30 min. period suggests application of shorter process times, for example up to 10 min.

EDS analysis results obtained for PLA and PLA–Cu^0^(t) (t = 10 min and 30 min) are presented in Table 3.

The content of carbon and oxygen components of poly(lactic acid) (PLA) (without of hydrogen) is similar to atomic “bulk” analysis of PLA (C = 50.0 and O = 44.4%). The surface modification of poly(lactic acid) samples using surface copper sputtering leads to appearance of copper, which contents rapidly increases during prolongation of sputtering (19.55% for PLA–Cu^0^(10), and 66.86 for PLA–Cu^0^(30)) and simultaneous substantial decrease of carbon (from 51.7% (PLA) to 42.33% (PLA–Cu^0^(10)) and 21.00 (PLA–Cu^0^(30)) and oxygen (from 48.33% (PLA) to 38.13% (PLA–Cu^0^(10)) and 12.14 (PLA–Cu^0^(30)) contents, respectively. This causes a subsequent change of the “surface molecular formula” (SMF) from C_3_O_2_ for PLA, to C_3_O_2_Cu_0.27_ for PLA–Cu^0^(10) and/or to C_3_O_1_._2_Cu_1.8_ for PLA–Cu^0^(30). These results suggest preferential deposition of copper atoms on the oxygens of carboxylate fragments of the lactide unit (–C(=O)–O–), as spatially more available due to flat structures of the carboxylic ester function.

### 3.2. ATR-FTIR Spectra

**Figure 4:**
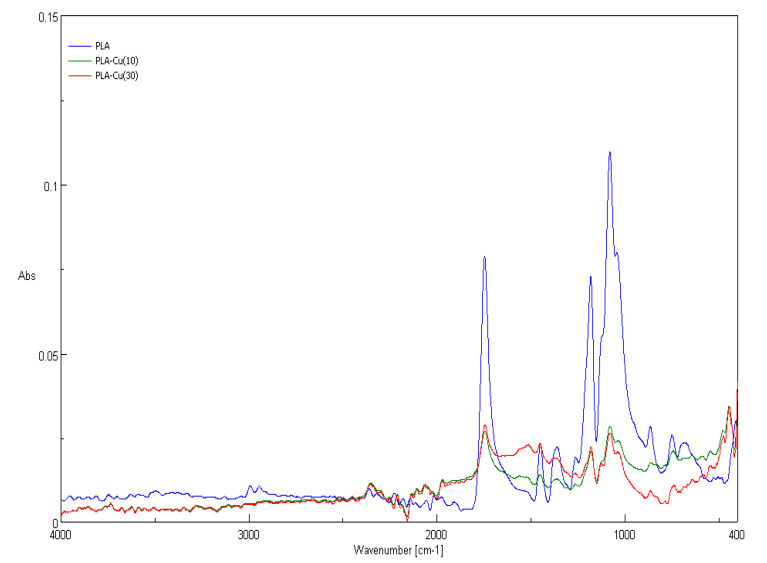
Attenuated total reflection Fourier transform infrared spectroscopy (ATR-FTIR) spectra of poly(lactic acid) non-woven and PLA–Cu^0^(10) and PLA–Cu^0^(30) composite.

The recorded ATR-FTIR spectra for PLA and PLA–Cu^0^(t) (t = 10 min, 30 min) composites are presented in Figure 4. Characteristic FTIR signals of the starting PLA and derived PLA–Cu(t) composites are summarized in Table 4.

The ATR-FTIR spectra of PLA as well as PLA–Cu^0^(t) composites are similar in a shape, exhibiting absorbance up to 0.12. Generally the major band intensities of PLA are much stronger than corresponding bands of PLA–Cu^0^(t) composites, which are almost identical.

The strongest bands of the presented spectra are: 1760 (0.09 (PLA) and 0.03 (PLA–Cu^0^(10)) identified as νC = O; 1270 (0.07(PLA) and 0.01 (PLA–Cu^0^(10))—identified as δCH + νCOC; 1215–1185 (0.08(PLA) and 0.03 (PLA–Cu^0^(10))—identified as ν_as_COC + r_as_CH_3_; 1130 (0.12 (PLA) and 0.03 (PLA–Cu^0^(10))—identified as r_as_CH_3_; 1100–1090 (0.06 (PLA) and 0.13 (PLA–Cu^0^(10))—identified as ν_s_ COC, and 1045 (0.08 (PLA) and 0.02 (PLA–Cu^0^(10)) identified as ν C-CH_3_.

The bands observed in the low-frequency region 740–695 cm^−1^ can be assigned to: δC = O (760–740 cm^−1^, absorbances 0.03 (PLA) and 0.02 (PLA–Cu^0^(10)) and γC = O (715–695 cm^−1^, absorbances 0.02 (PLA) and 0.01 (PLA–Cu^0^(10)).

The spectra of PLA–Cu^0^(t) offered noticeable differences, mainly at wavelength 1620 cm^−1^ (absorbances 0.00 (PLA), 0.013 (PLA–Cu^0^(10) and 0.021 (PLA–Cu^0^(30)). Also at 500–400 cm^−1^ absorbances derived from PLA–Cu^0^(t)composites are higher than for starting PLA. These differences can presumably have resulted from copper’s presence in the PLA and are similar to those obtained by Diaz-Visurraga et al. in a FTIR study of alginate-stabilized copper nanoparticles. [71]

### 3.3. UV-VIS Spectrometry and Determination of the Protective Properties against UV Radiation

**Figure 5:**
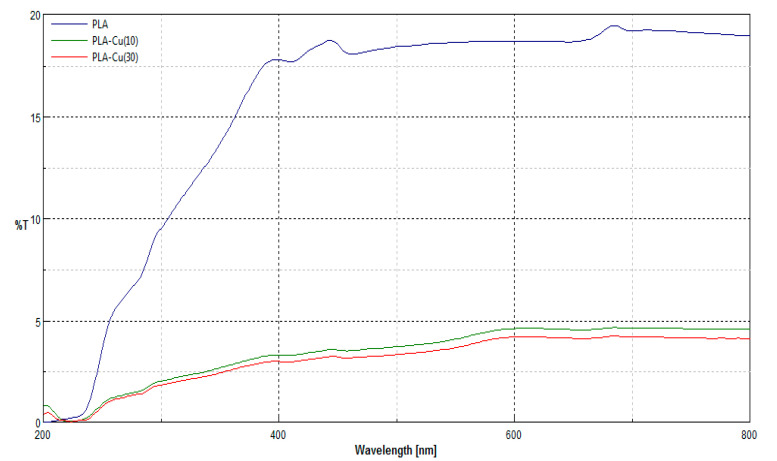
Transmittance spectra of PLA and PLA–Cu^0^(t) hybrids (PLA–Cu^0^(10) and PLA–Cu^0^(30)).

Transmittance spectra [%T] of PLA samples and PLA–Cu^0^(t) hybrids (PLA–Cu^0^(10) or PLA–Cu^0^(30)), recorded in the ranges *λ* = 200–800 nm is presented in Figure 5.

The transmittance (%T) spectra in the range *λ* = 200–800 nm of the modified PLA non-woven by magnetron sputtering show that the samples after modification reveal changes in the macrostructure expressed by a decrease in transmittance, the reduction in transmission is caused by an additional layer of copper on the surface of the samples. The transmittance spectra of modified samples (PLA–Cu^0^(10), PLA–Cu^0^(30)) had similar spectral characteristics and quite a similar level of transmittance in the entire spectral range when compared to control samples (without modification).

Table 5 compare average transmittance (T%) and calculated UPF values of modified samples (PLA–Cu^0^(10), PLA–Cu^0^(30)) with those non-modified. The transmittance (%T) spectra in the range *λ* = 290–400 nm. are presented in Figure 5.

Samples modified with copper obtain a UPF value >40, calculated on the basis of transmittance measurements for *λ* = 290–400 nm (according to Formula (1)). This result indicates that the modification performed imparts proper barrier properties against UV radiation according to PN-EN 13758–1:2002 [62].

### 3.4. Technical Parameters

Technical parameters of new composite materials were focused on tensile strength and filtration properties. Filtration properties expressed by the air permeability and were tested for starting poly(lactic acid) nonwoven and PLA–Cu^0^(t) composites. Results of filtration parameters are showed in Table 6. The results of tensile strength properties: relative elongation at maximum load [%] and durability for stretching [kN/m] of initial PLA samples and PLA–Cu^0^(t) composites are listed in Table 7.

The results of air flow resistance of modified polylactide show that 10 min magnetron sputtering modifications slightly decrease filtration parameters of PLA sample: PLA vs. PLA–Cu^0^(10): 458 vs. 437 at 50 Pa and 853 vs. 839 100 Pa, respectively but 30 min magnetron sputtering reduces filtration parameters: PLA vs. PLA–Cu^0^(30): 458 vs. 388 at 50 Pa and 853 vs. 778 at 100 Pa, respectively.

The listed tensile strength results of PLA and PLA–Cu^0^(t) composites show the ca. 11-fold increase for PLA–Cu^0^(10) (0.575 [kN/m) and 1.21-fold increase for PLA–Cu^0^(30) (0.071 [kN/m]), compared with unmodified PLA non-woven (0.05 [kN/m].

The results of relative elongation at maximum load [%] of poly(lactic acid) non-woven and PLA–Cu^0^(t) composites show ca. 2.5-fold increase of this parameter of PLA–Cu^0^(10) compared with PLA (PLA vs. PLA–Cu^0^(10): 11.98% vs. 28.75%) and ca. 4.5-fold decrease for PLA–Cu^0^(30) compared with PLA (PLA vs. PLA–Cu^0^(30): 11.98% vs. 2.65%). These results indicate that the sample PLA–Cu^0^(10) has a more beneficial flexibility and stronger structure compared with PLA. It is obvious also that very high loading of Cu on the PLA surface in PLA–Cu^0^(30) influenced negatively on the mechanical properties of this composite.

### 3.5. Flame Atomic Absorption Spectrometry

Determination of copper content in PLA–Cu^0^(t) composites was assessed by the Flame Atomic Absorption Spectrometry (FAAS) method [65] and listed in Table 8.

The results determination of copper content in poly(lactic acid) composite show that copper content in poly(lactic acid) composite samples depends on the applied magnetron sputtering deposition times (PLA–Cu^0^(10): 10 min-9.91 g/kg; PLA–Cu^0^(30): 30 min-27.89 g/kg) and the magnetron sputtering of copper deposition process is almost linear. The copper content in poly(lactic acid) composite indicate also that magnetron sputtering process is quite precise and distribution of copper in a composites bulk is uniform.

### 3.6. Antimicrobial Properties

#### Antibacterial Activity

The polylactide nonwoven (PLA) and polylactide-copper composites PLA–Cu^0^(t) were subjected to antimicrobial activity tests against Gram-negative *Escherichia coli* (ATCC11229) and Gram-positive *Staphylococcus aureus* (ATCC 6538) (Table 9).

**Figure 6:**
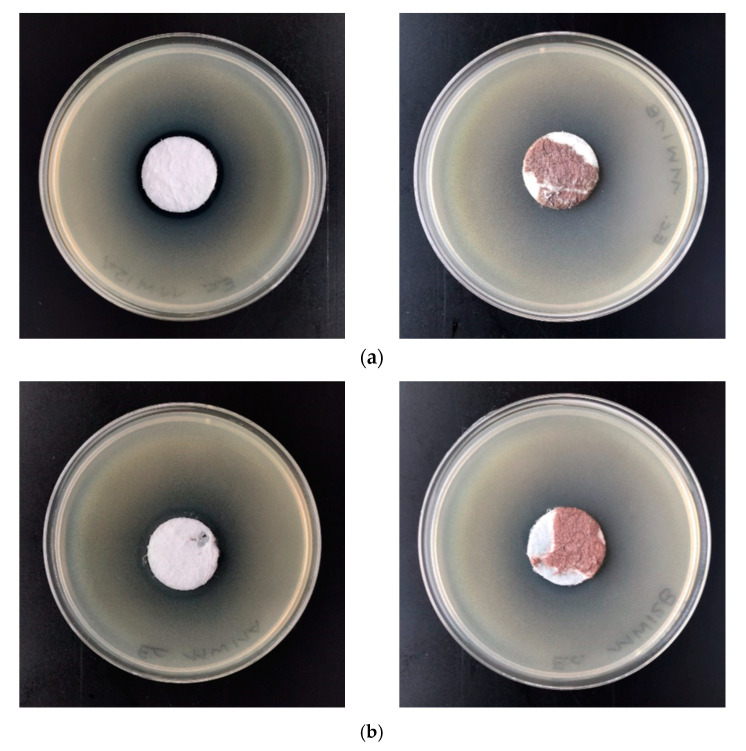
Inhibition zones of bacterial growth on Petri dishes, PLA–Cu^0^(10) sample: *Escherichia Coli* (**a**); *Staphylococcus aureus* (**b**).

Results of antimicrobial studies demonstrate antimicrobial protection against different bacterial species of new composites for *Escherichia coli* and *Staphylococcus aureus* (Table 8) according to standard: PN-EN ISO 20645:2006 [66]. Antimicrobial properties of composite samples expressed by strong visible inhibition zones of bacterial growth on inoculated agar Petri dishes (Figure 6) and no visible bacteria under the modified samples. Any antibacterial effect was observed for the unmodified sample (PLA). It is worth noting that 0.03 molar solutions of CuSO_4_ (2 mg/mL) are not bactericidal (Growth Inhibition Zone = 0) for several gram positive bacteria (e.g., *S. aureus*), gram negative bacteria (e.g., *E. coli*) bacteria as well as fungi species (e.g., *Candida* family) [72].

### 3.7. Antifungal Activity

**Figure 7:**
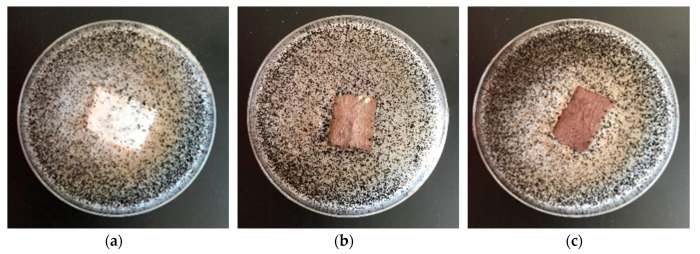
The sample antimicrobial activity tests against *Chaetomium globosum.* Inhibition properties of fungal growth on Petri dishes: (**a**)—PLA, (**b**)—PLA–Cu^0^(10); (**c**)—PLA–Cu^0^(30).

Results of antifungal tests against a *Chaetomium globosum* (ATCC 6205) of poly(lactide) nonwoven (PLA) and poly(lactide)-copper composites PLA–Cu^0^(t) are listed in Table 10 and Figure 7.

Modification of non-woven fabrics provide antifungal properties for *Chaetomium globosum*, expressed by no visible growth under the microscope (50× magnification). PLA non-woven fabrics without magnetron sputtering modification exhibit strong growth covering the surface of the control sample (Table 10, Figure 7).

## 4. Conclusions

The major contribution of this study was offering a new method for obtaining a multi-functional composite materials. The fabrication of new composite materials was performed by magnetron sputtering deposition of copper on the melt-blown poly(lactide) non-woven fabrics. The structure and mechanical properties of the obtained new composite products were characterized by FTIR spectrometry, UV/VIS transmittance, scanning electron microscopy (SEM), atomic absorption spectrometry with flame excitation (FAAS), tensile strength test and air permeability. The polylactide-copper composites were subjected to antimicrobial activity tests against: *Escherichia coli, Staphylococcus aureus, Chaetomium globosum*. The most important features of the new composite materials PLA–Cu^0^ are:

- eco-friendly, full biodegradable composite product;
- fabricated by clean and zero-waste process;
- improvement of technical parameters, including a tensile strength, air permeability and barrier properties against UV radiation of PLA–Cu^0^ synthesized in comparison with starting raw PLA non-woven fabrics;
- composite with potential antimicrobial properties.

The listed attributes of the PLA–Cu^0^ synthesized composites should find application in biomedical areas, and also as a microbiostatic material. Additionally, in the period when clinical waste are a major environmental burden, increasing interests should be paid to new biodegradable composite products fabricated by a clean process.

## Figures and Tables

**Table 1:** Representative application of antibacterial poly(lactide) (PLA) composites.

No	PLA Composite	Action/Application	Lit.
**1. PLA/ANTIBIOTICS/DRUGS**			
1.1	PLA/AMP	antibacterial activity (against *A.c.*, *E.f.*, *F.n.* and *P.g.*)	[9]
1.2	PLA/CHX	antibacterial activity against both G(+) and G(−) bacteria; the treatment of persistent infections in medicine and dentistry	[10,11]
1.3	PLA/CPX	potential application as a drug delivery system	[12,13]
1.4	PLA/DOX	chronic wound treatment	[14]
1.5	PLA/GM	delivery platforms with strong and timing controllable antibacterial properties (against *E.c.*)	[15]
1.6	PLA/OFL; PLA/VAN	antibacterial activity (against *E.c.* and MRSA), possible alternative drug against pathogenic bacterial strains of public health interest	[16]
1.7	PLA/Tric	antibacterial activity against G(+) and G(−)bacteria; a good osteoblast cell attachment on the composite scaffolds; a suitable composite for the bone tissue engineering and medical applications	[11,17,18,19]
1.8	PLA/Cur	antibacterial (against *S.a.*) and anticoagulant activity	[20]
**2. PLA/IONS/MNPS** [21,22,23,24,25,26,27]			
2.1	PLA/Ag+	antibacterial activity (against *S.a.*, *E.c.*); medical dressings	[28]
		Antibacterial properties (against *E.c.* and MRSA); potentantial bacterialwound-dressing materials	[29]
		Antibacterial polylactide materials (against *S.en.*) of significant potential in coating applications	[30]
2.2	PLA/CuSiO_3_	very good antibacterial activity (against *E.c.*, *S.a.*) and antifungal properties (against *C.a.*)	[31]
2.3	PLA/ZnO (surface or bulk deposited)	antimicrobial activity (against *S.a.*)	[32]
2.4	PLA/TiO_2_	no mammalian cell toxicity, bactericidal activity	[33]

Antibiotics/drugs: AMP—ampicillin; CHX—chlorhexidine; CPX—ciprofloxacin; Cur—curcumin; DOX—doxycycline; GM—gentamycin; OFL—ofloxacin; TCS (Tric)—triclosan; VAN—vancomycin. Bacteria: A.c.—*Actinomycetemcomitans;* E.f.—*Enterococcus faecalis;* E.c.—*Escherichia coli*; F.n.—*Fusobacterium nucleatum;* G(*+*)—Gram-positive bacteria, G(−) Gram-negative bacteria, respectively; MRSA—methicillin-resistant *Staphylococcus aureus*; P.g.—*Porphyromonas gingivalis*; S.a.—*Staphylococcus aureus*; S.en.—*Staphylococcus enterica.* Polymers/Nanoparticles/Additivities: AgNPs—silver nanoparticles; MNPS—metal nanoparticles; PL—polylactide; PDA—polydopamine.

**Table 2:** Melt-blown technique processing parameters applied for preparation of PLA non-woven.

Processing Parameters	
Polymer yields	6 g/min
Mass per unit area of nonwovens	102 g/m^2^
Air flow rate	7–8 m^3^/h
Head temperature	
Air heater temperature	260 °C
Temperature of the extruder in zone 3	260 °C
Temperature of the extruder in zone 2	245 °C
Temperature of the extruder in zone 1	195 °C

**Table 3:** Energy-dispersive spectroscopy (EDS) analysis results of PLA and PLA–Cu^0^(t).

	PLA (Calc.)			EDS Determinations							
				PLA		PLA–Cu^0^(10)^/a^			PLA–Cu^0^(30)^/a^		
Atom	C	H	O	C	O	C	O	Cu	C	O	Cu
%^/b^	50.0	5.6	44.4	51.7	48.33	42.33	38.13	19.55	21.00	12.14	66.86
Std. dev.^/c^				0.11	0.11	0.04	0.09	0.13	0.16	0.30	0.43
C:O:Cu/^d^ [%/%]	1:0.88:0			1: 0.93:0		1:0.90:0.46			1:0.58:3.18		
Ml^/e^	4.17	5.6	2.76	4.31	3.02	3.53	2.38	0.31	1.75	0.74	1.05
SMF^/f^	CH_1.3_O_0.66_; C_3_H_3.9_O_1.98_			C_3_O_2.1_		CO_0.67_Cu_0.09_; C_3_O_2_Cu_0_._27_			CO_0.4_Cu_0.6_; C_3_O_1.2_Cu_1.8_		

^a/^(t) = time of sputtering: 10 min or 30 min. ^b/^Percentage concentrations [g/110 g]; Mean value of 3 measurements. ^c/^Std. dev.—Standard deviation. ^d/^C:O:Cu ratios (%:%:%). ^e/^Ml—molar atoms contribution in the composite surface [mol/100 g]. ^f/^SMF—surface molecular formula.

**Table 4:** Characteristic FTIR bands determined for PLA non-woven and PLA–Cu^0^(10) composite.

PLA			PLA–Cu^0^(t)			
			PLA–Cu^0^(10)		PLA–Cu^0^(30)	
IR [ν/cm^−1^]	Intensity^/a^	Assignment [70]	IR [ν/cm^−1^]	Intensity^/a^	IR [ν/cm^−1^];	Intensity
2997	0.01	ν_as_ CH_3_	-	-	-	-
2947	0.01	ν_s_ CH_3_	-	-	2947	-
1760	0.08	ν C=O	1760	0.03	1760	0.03
1620	0.013		1620	0.014	1620	0.02
1452	0.02	δ_as_ CH_3_	1452	0.01	1452	0.02
1388–1368	0.02	δ_s_CH_3_	1348–1388	0.01	1348–1388	0.02
1368–1360	0.02	δ_1_ CH + δ_s_CH_3_	1368–1360	0.01	1368–1360	-
1270	0.02	δCH + νCOC	1270	0.01	1270	0.01
1215–1185	0.07	ν_as_COC + r_as_CH_3_	1215–1185	0.02	1215–1185	0.02
1130	0.05	r_as_CH_3_	1130	0.02	1130	0.02
1100–1090	0.11	ν_s_ COC	1100–1090	0.03	1100–1090	0.03
1045	0.08	ν C–CH_3_	1045	0.02	1045	0.02
875–860	0.03	νC–COO	875–860	0.02	875–860	0.01
760–740	0.03	δC=O	760–740	0.02	760–740	0.01
715–695	0.02	γC=O	715–695	-	715–695	-

Legend: Band assignment^/a^: as—asymmetric; δ—deformation; r—rocking; s—symmetric; sh—shoulder; γ—out-of-plane bending mode; ν—stretching vibration. Band intensity^/b^: Abs. were approximated to a second decimal place.

**Table 5:** Ultraviolet protection factor (UPF) values of non-woven fabric samples modified by magnetron sputtering.

Parameter	PLA	PLA–Cu^0^(10)	PLA–Cu^0^(30)
**UPF**	9.36	44.83	49.32
average %T, *λ* = 290–400 nm	13.5	2.66	2.42

The results have been measured in triplicate and presented as a mean value wit ± deviation approximately 2%.

**Table 6:** The air flow resistance of PLA and modified PLA–Cu^0^(t) sample according to: EN ISO 9237:1998 [63].

Parameter		PLA	PLA–Cu^0^(10)	PLA–Cu^0^(30)
Average air permeability ^a,b/^[mm/s], pressure decrease:	50 Pa	458	437	388
	100 Pa	853	839	778

^a/^The results have been measured in triplicate and presented as a mean value with deviation approximately ±2%. ^b/^The results were approximated to full numbers.

**Table 7:** Results of tensile strength test of PLA and hybrids PLA–Cu^0^(t), according to: EN ISO 10319:2015-08 [64].

Parameter	PLA	PLA–Cu^0^(10)	PLA–Cu^0^(30)
Tensile strength [kN/m] ^a,b/^	0.05	0.58	0.070
Relative elongationat maximum load [%] ^a,b/^	11.98	28.75	2.65

^a/^The results have been measured in triplicate and presented as a mean values with deviation approximately ±2%. ^b/^Values were approximated to a second decimal place.

**Table 8:** Results of determination of copper content in PLA composite samples.

Composite	Magnetron Sputtering Deposition Times of Copper [min.]	Cu Concentration	
		[g/kg]	[mol/kg]
PLA	-	0.004	
PLA–Cu^0^(10)	10	9.91	0.16
PLA–Cu^0^(30)	30	27.89	0.43

The results have been measured in triplicate and presented as a mean value with ± deviation approximately 2%.

**Table 9:** Results of tests on the antibacterial activity of polylactide nonwoven (PLA) and polylactide-copper composites (PLA–Cu^0^(10) and PLA–Cu^0^(30)) on the basis of standards EN-ISO 20645:2006 [66].

Sample Name	Bacterial Average Inhibition Zone(mm)	
	*Escherichia coli*	*Staphylococcus aureus*
PLA	0	0
PLA–Cu^0^(10)	2	1
PLA–Cu^0^(30)	2	1

Concentration of inoculum amount of live bacteria (bacterial suspension): *E. coli*: colony-forming units (CFU)/mL = 1.2 × 10^8^, *S. aureus*: CFU/mL = 1.7 × 10^8.^

**Table 10:** Results of antifungal activity tests of PLA composites on the basis of standard EN 14119: 2005 [69].

Sample Name	Fungal Average Inhibition Zone (mm)	
PLA	0	Visible growth on sample surface
PLA–Cu^0^(10)	1	No visible growth on sample surface
PLA–Cu^0^(30)	3	

Inoculum concentration (determined using a Thoma chamber), number of fungal spores in 1 mL [CFU/mL] = 2.2 × 10^6^.
